# Cortical porosity not superior to conventional densitometry in identifying hemodialysis patients with fragility fracture

**DOI:** 10.1371/journal.pone.0171873

**Published:** 2017-02-15

**Authors:** Bernhard Bielesz, Janina M. Patsch, Lukas Fischer, Marija Bojic, Wolfgang Winnicki, Michael Weber, Daniel Cejka

**Affiliations:** 1 Division of Nephrology and Dialysis, Department of Medicine III, Medical University of Vienna, Vienna, Austria; 2 Division of General Radiology and Pediatric Radiology, Department of Biomedical Imaging and Image-Guided Therapy, Vienna, Austria; 3 Computational Imaging Research (CIR) Laboratory, Department of Biomedical Imaging and Image-Guided Therapy, Vienna, Austria; 4 Software Competence Center Hagenberg, Hagenberg, Austria; 5 Department of Medicine III, Nephrology, Transplantation, Rheumatology, Geriatrics, Ordensklinikum Linz, Linz, Austria; University of Notre Dame, UNITED STATES

## Abstract

Hemodialysis (HD) patients face increased fracture risk, which is further associated with elevated risk of hospitalization and mortality. High-resolution peripheral computed tomography (HR-pQCT) has advanced our understanding of bone disease in chronic kidney disease by characterizing distinct changes in both the cortical and trabecular compartments. Increased cortical porosity (Ct.Po) has been shown to be associated with fracture in patients with osteopenia or in postmenopausal diabetic women. We tested whether the degree of Ct.Po identifies hemodialysis patients with prevalent fragility fractures in comparison to bone mineral density (BMD) assessed by dual X-ray absorptiometry (DXA). We performed a post-hoc analysis of a cross-sectional study in 76 prevalent hemodialysis patients. Markers of mineral metabolism, coronary calcification score, DXA-, and HR-pQCT-data were analyzed, and Ct.Po determined at radius and tibia. Ct.Po was significantly higher in patients with fracture but association was lost after adjusting for age and gender (tibia *p* = 0.228, radius *p* = 0.5). Instead, femoral (F) BMD neck area (*p* = 0.03), F T-score neck area (*p* = 0.03), radius (R) BMD (*p* = 0.03), R T-score (*p* = 0.03), and cortical HR-pQCT indices such as cortical area (Ct.Ar) (tibia: p = 0.01; radius: p = 0.02) and cortical thickness (Ct.Th) (tibia: *p* = 0.03; radius: *p* = 0.02) correctly classified patients with fragility fractures. Area under receiver operating characteristic curves (AUC) for Ct.Po (tibia AUC: 0.711; *p* = 0.01; radius AUC: 0.666; p = 0.04), Ct.Ar (tibia AUC: 0.832; *p*<0.001; radius AUC: 0.796; *p*<0.001), and F neck BMD (AUC: 0.758; *p* = 0.002) did not differ significantly among each other. In conclusion, measuring Ct.Po is not superior to BMD determined by DXA for identification of HD patients with fragility fracture.

## Introduction

Fracture risk in chronic kidney disease increases with declining renal function [1]. Moreover, a fracture in hemodialysis patients is associated with elevated risk of hospitalization and mortality [2]. Renal osteodystrophy affects the majority of patients with advanced chronic kidney disease (CKD) and is one feature of chronic kidney disease—mineral bone disorder (CKD-MBD) syndrome [3].

Chronic kidney disease patients suffer from decreased cortical and trabecular bone mineral density, cortical thinning, and disturbed trabecular microarchitecture [4–7]. High-resolution peripheral quantitative computed tomography (HR-pQCT) revealed predominant decreases in bone mineral density in the cortical compartment, decreased radial cortical area and cortical thickness, and elevations in cortical porosity of both tibia and radius [8, 9]. Changes in cortical porosity have been shown to have a significant impact on the resistance to fracture [10–13].

There are numerous studies, both retrospective and prospective, on the value of DXA on the assessment of fracture risk in CKD [14–21]. Jamal et al. [22] performed pQCT measurements of the radius in dialysis patients and reported decreased cortical density, cortical area, and cortical thickness of the radius to be associated with fractures. In a previous study by our group, HR-pQCT parameters at the tibia discriminated dialysis patients with fractures from those without fractures [4].

Although higher cortical porosity as a measure of deteriorated cortical bone architecture is well recognized in chronic kidney disease throughout all stages, the presumed clinical value of this parameter is less well defined in this patient population. Based on our hypothesis that fracture patients exhibit higher cortical porosity, we investigated whether measuring cortical porosity identifies patients with prevalent low-trauma fractures. We further compared this parameter to common cortical HR-pQCT and DXA-derived parameters for their ability to identify fragility fractures in a representative Mid-European dialysis patient cohort.

## Materials and methods

The study protocol was approved by the ethics committee of the Medical University of Vienna and adhered to the Declaration of Helsinki. All hemodialysis patients from our department were invited to participate. Exclusion criteria were age under 18 and pregnancy. None of the participants were on antiresorptive bone medication. Written informed consent was obtained from all participating patients. We performed a post-hoc analysis of a cross-sectional study with 76 patients on maintenance hemodialysis who were recruited between 2008 and 2010. Results of subsets of this cohort have been published previously [4, 23]. Patient charts were reviewed for demographic and laboratory data and for history of low-trauma fractures of any age at the time of HR-pQCT measurement. Low-trauma resulting in fragility fractures was defined as a fall from standing-height or lower. In addition, lateral spine x-rays and chest x-rays were screened for previously unrecognized fractures at the time of HR-pQCT measurement [24]. Survival data until 2014 were retrieved from the Austrian Dialysis and Transplant Registry (ÖDTR) [25].

### HR-pQCT

HR-pQCT was performed on all patients at both tibia and radius on an XtremeCT scanner from Scanco Medical, Switzerland, and analyzed as described previously [4, 26]. The tibia of the non-dominant leg and the radius of the arm that was either devoid of or carried a dysfunctional arteriovenous fistula were analyzed. Only apparently intact areas without history of fracture were used for the examination. 110 CT slices were obtained at each site, from which a 9-mm long 3D-volume was reconstructed. Image analysis was performed as outlined by Burghardt et al. (reviewed in [27]). Periosteal boundary and cortical and trabecular compartments were semi-automatically contoured as described [28]. A threshold-based algorithm and edge enhancement distinguished trabecular structures from cortical bone [29]. Calculation of parameters was performed as described [30]. Dcomp represents apparent cortical density in mg/cm^3^ hydroxyapatite (HA), Ct.Ar cortical area in mm^2^, Tb.Ar trabecular area in mm^2^, D100 average bone density in mg/cm^3^ HA of both cortical and trabecular compartment, Ct.Th cortical thickness (the mean cortical volume divided by the outer surface) in mm, Dtrab trabecular density in mg HA/cm^3^, BV/TV (%) bone volume fraction in % [100 x (Dtrab in mg HA/cm^3^)/1200 mg HA/cm^3^], TbN trabecular number per mm, TbTh trabecular thickness [(BV/TV)/TbN], and Tb1/NSD (mm) trabecular separation. Contouring of the cortical compartment for determination of cortical porosity was manually corrected when overt misalignment with the trabecular compartment occurred [31, 32]. Cortical porosity was calculated as ratio of cortical pore volume (empty space) to total cortical volume (sum of empty space and mineralized matrix).

### Coronary calcium scoring

Assessment of calcium burden was performed on an ECG-gated 16-slice scanner (Siemens Somatom16, Siemens, Forchheim, Germany) [33]. All coronary artery calcification (CAC) data sets were analyzed by a single technician with more than 5 years of experience in cardiac CT imaging using a commercially available software package (“Syngo CaScore”; Siemens Healthcare, Forchheim, Germany). Patients with coronary artery stents were excluded from analysis as the stent graft would have yielded false-high calcification scores.

### Dual-energy X-ray Absorptiometry (DXA)

Bone mineral density measurements (BMD) were performed with dual-energy X-ray absorptiometry on a QDR-4500 scanner (Hologic, Waltham, MA), using the manufacturer's recommended standard procedures [33].

### Laboratory parameters

Bone alkaline phosphatase (bAP) was measured using an immunosorbent enzyme-linked assay (Metra Biosystems, Behring Diagnostic, Eschborn, Germany). C-telopeptide pyridinoline cross-links of type I collagen (CTX, CrossLaps) and 25-hydroxy-cholecalciferol (25(OH)D) were measured by electro-chemiluminescence (Modular and Elecsys Systems, Roche, Switzerland). Calcitriol (1,25(OH)_2_D) was analyzed after chromatographic separation by radioimmunoassay (DiaSorin). Parathyroid hormone (PTH) was measured using a second generation assay (Elecsys PTH intact, Roche, Switzerland). Calcium and phosphate were quantified by routine clinical chemistry analyzers (Olympus, Japan). All measurements were performed according to Good Laboratory Practice (GLP) standards at the Institute of Laboratory Medicine of the General Hospital in Vienna.

### Statistics

All statistical computations were performed using IBM SPSS Statistics for Windows (Version 23). Metric data were described using mean and SD or as otherwise indicated. Spearman’s rank correlation was used to assess bivariate correlations between cortical porosity (separately for tibia and radius) and age in addition to coronary calcium score. Point biserial correlations were used for the correlation between cortical porosity and fracture (yes/no) as well as mortality. In case of significant results associations were adjusted for age and sex using either multiple binary logistic regression (for fractures and mortality) or multiple linear regression (for coronary calcification). We performed factor analysis to avoid multiple testing and multiplicity corrections in a cohort of limited sample size and of in large parts highly correlated parameters. Receiver operating characteristic (ROC) analyses and subsequent De-Long testing were performed to compare area under the curves (AUC) of indicated parameters to discriminate patients with and without fractures. A p-value equal or below 0.05 was assumed to indicate significant results.

Radius Ct.Po could not be analyzed in one patient due to insufficient quality of acquisition data. One patient’s set of measurements of the tibia had to be removed because of preceding paraplegia for many years due to a childhood accident. Coronary calcium burden was unavailable in 15 patients, and DXA BMD measurements of the radius were available only in a subset of 51 patients. Therefore, we excluded radius BMD measurements in the ROC analysis.

## Results

Demographic baseline data stratified according to fracture status are shown in Table 1. Patients with fractures were older with higher female proportion, and also had lower serum phosphate levels. 22 patients (29%) with current or history of low-trauma fracture were observed at the time of analysis. We observed 3 rib fractures, 5 femoral neck fractures, and 19 vertebral fractures; 5 patients had 2 documented fractures. We did not observe significant between group differences with respect to diabetes, PTH, history of corticoid use, use of active Vitamin D medication, or Cinacalcet.

**Table 1:** Patient demographic data.

	No Fracture	Fracture	*p*
N	54	22	
Age (years median)	55 ± 12.7	71 ± 12.7	**<0.001**
Sex F/M	17/37	15/7	**0.005**
BMI (kg/m^2^ median) (75)	25.3 (22.8–27.9)	22.6 (20.1–26.5)	0.08
Dialysis (months median) (72)	33.8 (12.2–69.4)	50.5 (14.7–65)	0.55
bAP (ng/mL median) (62)	24.1 (15.1–30.1)	24.1 (15–35.5)	0.84
PTH (pg/mL median) (74)	206 (158–339)	217 (87–346)	0.51
Calcium (mmol/L) (75)	2.22 ± 0.16	2.24 ± 0.15	0.75
Phosphate (mmol/L) (75)	1.9 ± 0.42	1.68 ± 0.28	**0.01**
CTX (ng/mL median) (49)	1.89 (1.35–2.76)	1.72 (1.3–2.1)	0.33
25(OH)D (nmol/L median) (72)	38.1 (32.1–50.8)	36 (23.4–42.4)	0.33
1,25(OH)_2_D (pg/mL median) (73)	9.6 (6.7–15.8)	13.9 (8.4–17.5)	0.24
Vitamin K antagonist (%) (72)	1.9	5	0.481
Cinacalcet (%)	13	22.7	0.312
Smoker (%) (65)	30.4	10.5	0.119
Diabetes (%) (75)	33.3	28.6	0.787
History of glucocorticoid use (%) (75)	29.6	14.3	0.241
Active Vitamin D medication (%) (75)	49.1	59.1	0.458
CT-scan coronary calcification (%) (61)	69.6	80	0.524
Hip fractures (%) (70)	0	22.7	n.a
Deceased by the time of data analysis (%)	29.6	72.7	**0.001**

bAP: bone specific alkaline phosphatase; PTH: parathyroid hormone; CTX: C-terminal telopeptide; BMI: body mass index; 25(OH)D: 25-hydroxy-Vitamin D3; 1,25(OH)_2_D: 1, 25-hydroxy-Vitamin D3; shown are mean and standard deviation or median and interquartile range (25/75). Numbers in parentheses indicate sample number if different from total N. Reference ranges: bAP—men: 6–30 ng/mL and women: 6–26 ng/mL; PTH—15–65 pg/mL; CTX—men: 0.08–0.35 ng/mL and women: 0.09–0.44 ng/mL; 25(OH)D—28–107 nmol/L; 1,25(OH)_2_D—25–66 pg/mL; calcium—2.2–2.65 mmol/L; phosphate: 0.91–1.45 mmol/L.

Two thirds of the patients had documented coronary artery calcification of varying severity. Although there was no association of the degree of coronary calcification with fractures, we noted some univariate correlation of Ct.Po with coronary calcification score (tibia: *r* = 0.342, *p* = 0.007; radius: *r* = 0.366, *p* = 0.004) that did not persist after adjusting for age and gender in logistic regression (tibia: *p* = 0.98; radius: *p* = 0.3). By the time of data analysis approximately twice as many patients had died in the fracture group which is also reflected by positive associations of Ct.Po with mortality in unadjusted (tibia: *r* = 0.318, *p* = 0.005; radius: *r* = 0.31, *p* = 0.007), but not in adjusted analysis (tibia: *p* = 0.7; radius: *p* = 0.37).

**Fig 1:**
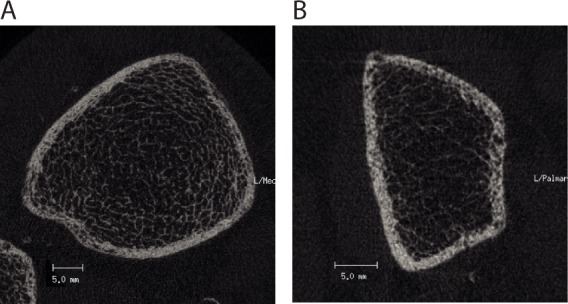
HR-pQCT sections of tibia and radius. Representative images of tibia (A) and radius (B) from hemodialysis patients with high cortical porosity. (A): tBV/TV (%): 14.6, Ct.Po (%): 11.54; (B): tBV/TV (%): 7.7, Ct.Po (%): 11.49.

Table 2 shows a selection of cortical and trabecular parameters derived from peripheral HR-pQCT analysis and bone density measurements from dual energy X-ray absorptiometry stratified according to fracture status and sex. There was a trend to higher Ct.Po in men (tibia: *r* = 0.159, *p* = 0.17; radius: *r* = 0.182, *p* = 0.12) and higher values were observed in patients with fracture compared to patients without fractures in both genders. Fig 1 shows representative images of tibial (A) and radial (B) bone in a dialysis patient with high cortical porosity.

**Table 2:** Patient HR-pQCT morphometric data stratified according to fracture status and gender.

	No fracture		Fracture	
Tibia	Female	Male	Female	Male
D100 (mg/cm^3^) (75)	248 ± 57.3	250 ± 60	180 ± 45.7	239 ± 83.7
Tb.Ar (mm^2^) (75)	554 ± 108	718 ± 112	597 ± 118	734 ± 224
Dtrab (mg HA/cm^3^) (75)	133 ± 40.9	146 ± 43.3	108 ± 40.6	151 ± 29.5
tTbN (1/mm) (75)	1.53 ± 0.371	1.82 ± 0.461	1.35 ± 0.428	1.85 ± 0.307
tTb.Sp (mm) (75)	0.62 ± 0.181	0.505 ± 0.142	0.688 ± 0.218	0.483 ± 0.082
Tb1/NSD (mm) (75)	0.331 ± 0.163	0.252 ± 0.114	0.427 ± 0.28	0.229 ± 0.057
tTb.Th (mm) (75)	0.072 ± 0.012	0.067 ± 0.016	0.069 ± 0.024	0.069 ± 0.012
Ct.Ar (mm^2^) (75)	94.8 ± 21.7	119 ± 32.6	58.6 ± 26	87.6 ± 47.6
Dcomp (mg HA/cm^3^) (75)	834 ± 71	804 ± 65.4	712 ± 103	726 ± 125
Ct.Th (mm) (75)	0.951 ± 0.259	1.04 ± 0.297	0.58 ± 0.269	0.794 ± 0.501
tBV/TV (%) (75)	11.1 ± 3.4	12.2 ± 3.6	9 ± 3.4	12.6 ± 2.5
Ct.Po (%) (75)	5.53 ± 3.26	8.46 ± 3.24	9.73 ± 4.04	10.6 ± 2.98
Radius				
D100 (mg/cm^3^)	279 ± 82.6	279 ± 93.4	217 ± 56.2	265 ± 72.6
Tb.Ar (mm^2^)	195 ± 47	282 ± 74.1	201 ± 42.3	272 ± 52.1
Dtrab (mg HA/cm^3^)	114 ± 45.8	148 ± 50.6	101 ± 50.3	152 ± 39.7
tTbN (/mm)	1.59 ± 0.357	1.87 ± 0.389	1.36 ± 0.523	1.71 ± 0.789
tTb.Sp (mm) (75)	0.599 ± 0.151	0.487 ± 0.146	0.692 ± 0.262	0.452 ± 0.099
Tb1/NSD (mm) (75)	0.283 ± 0.102	0.254 ± 0.164	0.438 ± 0.252	0.235 ± 0.125
tTb.Th (mm)	0.058 ± 0.012	0.065 ± 0.016	0.061 ± 0.014	0.063 ± 0.009
Ct.Ar (mm^2^)	46.8 ± 14.8	54.3 ± 20.1	30.3 ± 9.33	44.2 ± 17.9
Dcomp (mg HA/cm^3^)	845 ± 84.7	798 ± 96.5	748 ± 98.1	760 ± 104
Ct.Th (mm)	0.721 ± 0.25	0.686 ± 0.287	0.466 ± 0.168	0.554 ± 0.24
tBV/TV (%)	9.5 ± 3.8	12.3 ± 4.2	8.2 ± 4.2	12.7 ± 3.3
Ct.Po (%) (75)	2.02 ±1.21	3.61 ± 2.38	4.26 ± 2.75	4.72 ± 2.3
DXA				
F BMD total (g/cm^2^) (70)	0.803 ± 0.151	0.856 ± 0.165	0.664 ± 0.128	0.772 ± 0.109
F T total (69)	-1.15 ± 1.23	-1.17 ± 1.13	-2.29 ± 1.05	-1.75 ± 0.734
F BMD neck (g/cm^2^) (71)	0.682 ± 0.148	0.697 ± 0.157	0.557 ±0.094	0.572 ± 0.095
F T neck (71)	-1.5 ± 1.33	-1.76 ± 1.21	-2.63 ± 0.854	-2.65 ± 0.718
LS BMD (g/cm^2^) (69)	0.902 ± 0.148	0.993 ± 0.172	0.85 ± 0.164	0.968 ± 0.191
LS T (69)	-1.37 ± 1.46	-0.897 ± 1.56	-1.79 ± 1.48	-1.12 ± 1.76
R BMD (g/cm^2^) (51)	0.47 ± 0.062	0.545 ± 0.094	0.365 ± 0.064	0.475 ± 0.086
R T (51)	-2.02 ± 1.16	-2.73 ± 1.8	-3.94 ± 1.22	-4.1 ± 1.64

Ct.Ar: cortical area; Tb.Ar: trabecular area; Dtrab: trabecular density; TbN: trabecular number; tTb.Sp: trabecular separation; Tb1/NSD: standard deviation of 1/Tb.N; tTb.Th: trabecular thickness; D100: average bone density; Dcomp: apparent cortical bone density; Ct.Th: cortical thickness; tBV/TV: trabecular bone volume/tissue volume; Ct.Po: cortical porosity; BMD: bone mineral density; FN: femoral neck; LS: lumbar spine; R: radius; T: T-score. Numbers in parentheses indicate sample number if different from total N.

**Fig 2:**
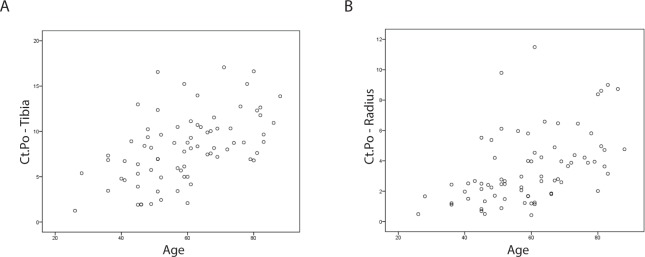
Association of Ct.Po and age in hemodialysis patients in the tibia (A) and radius (B).

There was a strong correlation of Ct.Po with age (tibia: *r* = 0.524, p<0.001; radius: *r* = 0.541, *p*<0.001) (Fig 2A and 2B). We observed associations of Ct.Po with the presence of documented fractures at both sites in unadjusted analysis. However, this association did not persist after adjusting for age and gender in logistic regression analysis (Table 3).

**Table 3:** Presence of fracture discriminated by DXA and cortical HR-pQCT parameters.

	Beta	p-value	Beta*	p-value*
Tibia				
Ct.Po	0.193	**0.011**	0.126	0.228
Cr.Ar	-0.042	**<0.001**	-0.028	**0.013**
D100	-0.014	**0.020**	-0.014	**0.020**
Dcomp	-0.013	**<0.001**	-0.009	**0.030**
Ct.Th	-3.714	**<0.001**	-2.380	**0.027**
Radius				
Ct.Po	0.221	**0.041**	0.112	0.500
Cr.Ar	-0.067	**0.001**	-0.053	**0.023**
D100	-0.008	**0.034**	-0.009	0.061
Dcomp	-0.007	**0.019**	-0.005	0.161
Ct.Th	-3.653	**0.005**	-3.695	**0.017**
DXA				
F total BMD	-5.417	**0.007**	-4.781	0.063
F total T	-0.657	**0.012**	-0.621	0.060
F neck BMD	-7.298	**0.008**	-6.643	**0.032**
F neck T	-0.746	**0.032**	-0.740	**0.032**
LS BMD	-3.031	0.096	-3.332	0.162
LS T	-0.254	0.192	-0.328	0.187
R BMD	-16.112	**0.001**	-14.037	**0.029**
R T	-0.622	**0.005**	-0.708	**0.033**

Significant parameters in the unadjusted model were used in a multiple regression model adjusted for age and gender; Ct.Po: cortical porosity; Ct.Ar: cortical area; D100: average bone density; Dcomp: apparent cortical bone density; Ct.Th: cortical thickness; BMD: bone mineral density; F: femur; LS: lumbar spine; R: radius; T: T-score.

*…adjusted model for age and gender.

To relate these findings to the performance of other DXA- and HR-pQCT-based parameters, we analyzed DXA-derived BMD data and cortical HR-pQCT parameters. Dcomp, Ct.Ar, D100, and Ct.Th of tibia and Ct.Ar and Ct.Th of radius and F BMD neck, F T neck, R BMD, and R T remained significantly associated with low-trauma fractures after adjustment for age and gender (Table 3).

As Ct.Po lost its association in adjusted analysis, we performed a post-hoc power analysis. Based on the distribution of patients with and without fracture in our cohort, a total sample size of 1532 (no fracture: 1021; fracture: 511) would be required to reach statistically significant differences for Ct.Po at the tibia (power: 80%, p<0.05, two-sided) between these two groups. For Ct.Po at the radius approximately 55000 patients would be necessary.

**Fig 3:**
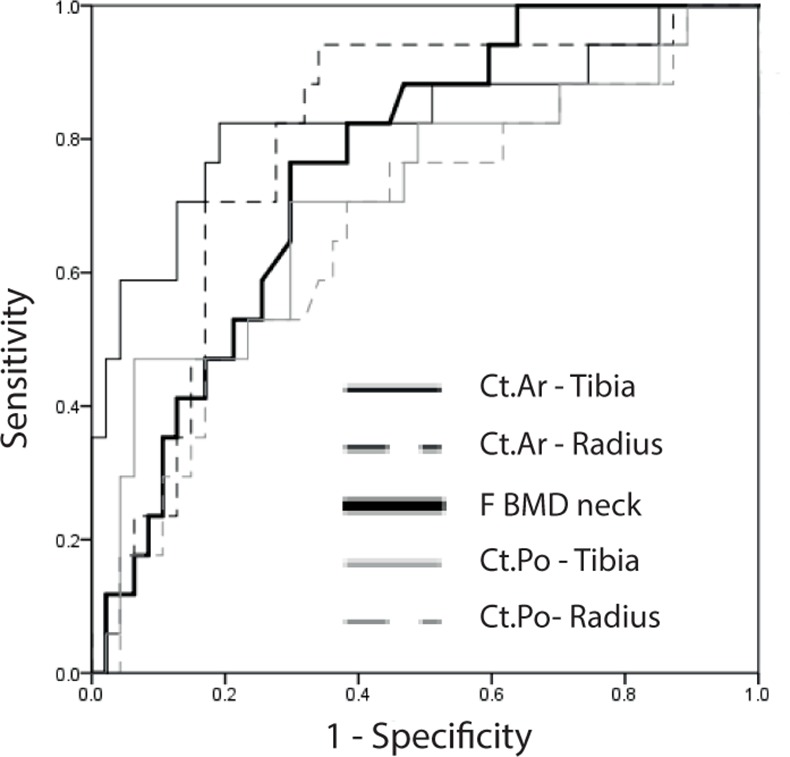
ROC curves: F neck BMD, Ct.Ar, and Ct.Po of tibia and radius discriminating fracture. Higher area under the curve (AUC) indicates a more favorable sensitivity-specificity profile. The ROC curves of Ct.Po (radius and tibia) are compared to the ROC curves with the highest AUC among HR-pQCT parameters (Ct.Ar of radius and tibia) and densitometric parameters (F neck BMD). For exact AUC values see Table 4; De-Long comparisons of ROC curves: Tibia: Ct.Po vs. Ct.Ar: *p* = 0.19; Ct.Po vs. F BMD neck: *p* = 0.6; Ct.Ar vs. F BMD neck: *p* = 0.27; Radius: Ct.Po vs. Ct.Ar: *p* = 0.2; Ct.Po vs. F BMD neck: *p* = 0.34; Ct.Ar vs. F BMD neck: *p* = 0.59; ROC: receiver-operating-characteristic; Ct.Po: cortical porosity; Ct.Ar: cortical area; BMD: bone mineral density; F: femur.

To assess sensitivity-specificity profiles of the above tested parameters, we calculated receiver-operating-characteristic (ROC) curves (Table 4). All tested parameters except lumbar spine (LS) BMD and LS T showed areas under the curve (AUC) which significantly differed from the line of equality (AUC of 0.5). None of the analyzed indices including Ct.Po differed significantly from the AUC of BMD in the femoral neck region (Fig 3 and Table 4).

**Table 4:** ROC areas under the curve (AUC) for HR-pQCT parameters including Ct.Po.

	AUC	*p*-value*
Tibia		
Ct.Po	0.711	**0.010**
Cr.Ar	0.832	**<0.001**
Ct.Th	0.797	**<0.000**
D100	0.735	**0.004**
Dcomp	0.791	**<0.001**
Radius		** **
Ct.Po	0.666	**0.043**
Cr.Ar	0.796	**<0.001**
Ct.Th	0.749	**0.002**
D100	0.683	**0.026**
Dcomp	0.697	**0.017**
DXA		** **
F total BMD	0.749	**0.002**
F total T	0.734	**0.004**
F neck BMD	0.758	**0.002**
F neck T	0.732	**0.005**
LS BMD	0.649	0.070
LS T	0.606	0.196

*…vs. line of equality (AUC = 0.5); ROC: receiver-operating-characteristic; Ct.Po: cortical porosity; Ct.Ar: cortical area; D100: average bone density; Dcomp: apparent cortical bone density; Ct.Th: cortical thickness; BMD: bone mineral density; F: femur; LS: lumbar spine; T: T-score.

## Discussion

To the best of our knowledge, this is the first study to investigate the association between cortical porosity and fractures in patients with dialysis-dependent end-stage renal disease and to subsequently relate this parameter to other HR-pQCT indices and DXA parameters. We found that Ct.Po is higher in dialysis patients with prevalent fragility fractures as indicated by univariate correlation. However, significance was lost after adjusting for significant confounders. Rather, cortical area and other cortical HR-pQCT- and DXA-derived parameters discriminated HD patients with low-trauma fractures in the adjusted analysis.

The lack of a direct association between Ct.Po and fracture status is in contrast to findings reported in other patient populations: Ct.Po has been shown to identify postmenopausal diabetic women with fragility fractures [12]. In another study Ct.Po discriminated women with fractures but only in the subgroup with osteopenia and not in the group with overt osteoporosis [10]. This might indicate that increased Ct.Po exerts a detrimental effect on cortical bone strength before substantial decreases in bone mass occur [13]. However, in patients with severely reduced bone mass with cortical thinning such as the dialysis population studied here, the relative contribution of Ct.Po to mechanical stability of bone might become smaller. In addition, it must be recognized that the pathophysiology of renal osteodystrophy as part of CKD-MBD syndrome, which also significantly influences bone quality, differs substantially from osteopenia and osteoporosis [34].

We confirm previous reports from non-CKD cohorts by finding a high association between cortical porosity and age in dialysis patients as well [32, 35, 36]. We also observed some significant associations between Ct.Po with CAC and mortality in univariate analyses, which were most likely mediated by age as they did not prevail in multivariate analysis. However, these analyses were exploratory in nature; larger studies would be required to ascertain as to whether Ct.Po associates with CAC or mortality independently of age.

HD patients with fracture are at substantially increased risk for adverse outcomes as described in a recent large observational study [2]. Not only is the overall fracture rate higher in HD patients compared to the general population, but also rates of death and re-hospitalization increase after a fracture event [2]. Clinically, DXA is currently the most commonly used technique to determine BMD and assess fracture risk in osteoporosis patients. There is some debate as to the role of DXA in CKD, HD, and renal transplant patients in assessing fracture risk, which has been addressed by a substantial number of studies [14–16, 18, 20].

BMD was prospectively shown to predict fractures in females with low parathyroid hormone (PTH); it also discriminated prevalent spine fractures in HD patients [17]. According to a prospective study of CKD patients not on dialysis both BMD and HR-pQCT indices indicated risk for future fracture [19]. This is also consistent with findings from a large cohort of elderly participants with and without CKD [21].

There is considerable interest in a potential diagnostic role of HR-pQCT in renal osteodystrophy [7–9, 22, 37]. We have previously shown that HR-pQCT parameters of the tibia in this prevalent hemodialysis cohort discriminate HD patients with fragility fractures [4]. Interestingly, Ct.Th at the radius reached only borderline significance (p = 0.06) in the preceding report. In this cohort, which contains additional patients and fracture data, we observed significant correlations of Ct.Ar and Ct.Th with fracture status at both sites (Table 3).

Our study has limitations. A significant number of patients suffered from long-standing renal-insufficiency and secondary hyperparathyroidism. We did not perform bone-biopsies that could further delineate the turnover-state of the bones, which likely has major impact on cortical bone architecture over time. However, as this information is rarely available in clinical routine, our findings are well applicable in an unselected dialysis population of the kind under study.

Cortical thinning as well as changes in the trabecular compartment might result in unusual anatomic circumstances at the endocortical border that are not found to this extent in other populations studied. We performed an automated cortex-contouring process and minimized manual intervention, except when overt misalignments occurred (inner contour lines falsely extending far into trabecular bone). However, endocortical bone resorption and increased porosity close to the endocortical area might lead to underestimation of Ct.Po when the original endocortical border is no longer clearly identified.

We do not have information regarding when the fracture occurred in relation to the time of HR-pQCT measurement when fracture data were collected. As we screened all available radiographic material of the study population, a significant proportion of patients were likely unaware of the fracture or when it was acquired. Owing to this and the retrospective nature of this study, our study precludes any conclusion as to whether increased cortical porosity was causative for the observed fractures. However, although measurement of Ct.Po did not outperform conventional densitometry, our data are well in agreement of an important physiological role of Ct.Po in fracture resistance.

Although our post-hoc power analysis showed that a far greater patient number would be necessary to demonstrate possible associations of Ct.Po with low-trauma fracture, sample size was large enough to readily confirm such associations with F BMD neck, Ct.Ar, and Ct.Th. This argues against a superior clinical role for Ct.Po in fracture discrimination in hemodialysis patients. This is further corroborated by the ROC analysis, where we assessed how Ct.Po, cortical HR-pQCT, and DXA-derived parameters compare in their ability to correctly classify fragility fractures in HD patients. We observed only small differences between AUCs of Ct.Po and F BMD neck that did not reach statistical significance and are also unlikely to reflect clinical relevance. It merits further study whether restricting HR-pQCT/Ct.Po analysis to patients of a certain age range or according to clinical pre-assessment of fracture risk would alter these results.

In conclusion, cortical porosity of the tibia or radius assessed by HR-pQCT is of no added value in comparison to BMD measurement by DXA for identification of prevalent HD patients with fragility fracture.

## Supporting Information

S1 Table Raw data file. (XLSX)
